# Successful kidney transplantation during anakinra treatment without complications

**DOI:** 10.1186/1546-0096-13-S1-P193

**Published:** 2015-09-28

**Authors:** C Mulders-Manders, F Molenaar, M Baas, A Simon

**Affiliations:** 1 Radboud University Medical Center, Internal Medicine, Nijmegen, the Netherlands; 2 Radboud University Medical Center, Nijmegen Center for Immunodeficiency and Autoinflammaton (NCIA), Nijmegen, the Netherlands; 3 University Medical Center, Nephrology, Utrecht, the Netherlands; 4 Radboud University Medical Center, Nephrology, Nijmegen, the Netherlands

## 

Although the incidence of end stage renal failure in patients with autoinflammatory diseases has decreased since the introduction of the interleukin-1 (IL-1)-receptor antagonist anakinra, it is still relatively common. Cessation of anti-IL-1 therapy during and after kidney transplantation will not be possible in most patients because of the risk of recurrence of inflammation. The effect of continued anti-IL-1 therapy on transplantation-related complications is currently unknown.

Recently, we have performed kidney transplantation in two patients while on anakinra therapy. The first patient is a 70 year old male, who suffered from febrile episodes since 2004 and was diagnosed with adult onset Still's disease in 2010. He had been using anakinra ever since. Chronic renal insufficiency due to thrombotic microangiopathy had been present since 2005. In September 2014 he received a kidney transplant of a living related donor, under standard immunosuppression (tacrolimus, prednisone and mycophenolate mofetil (MMF)), while anakinra was continued. Kidney function improved rapidly and he could be discharged from the hospital 7 days post transplantation. Until March 2015, he has been readmitted two times: once for a single day because of drainage of a wound abscess, and for 5 days 1 year post-transplantation because of sepsis with unknown cause. He now has adequate and stable transplant function.

The other patient is a 20 year old woman with mutation-negative Chronic Infantile Neurologic Cutaneous Articular (CINCA) syndrome. She had been using anakinra since 2004, which was switched to canakinumab in 2012. She was known with chronic renal insufficiency with proteinuria, probably due to recurrent urinary tract infections, vesico-urethral reflux and chronic use of NSAIDs since 2009. Kidney biopsy was contra-indicated. Because of rapidly progressive renal function loss, she received a pre-emptive kidney transplant of a healthy related donor in September 2014, under standard immunosuppression. Canakinumab was switched to anakinra just before transplantation, because of shorter half-life, making it easier to cease in case of complications. Kidney function increased rapidly and she could be discharged 6 days post-transplantation. She had been readmitted a single time for two days because of a primo-EBV and influenza infection in December 2014. She now has normal kidney function.

In these two cases we did not find an increase of transplantation-related complications or post-transplantation infectious complications, when kidney transplantation with a standard immunosuppresive regimen was performed during anakinra therapy. It is important to adapt the dosing of anakinra to the kidney function before and after transplantation.

Written informated consent for publication of their clinical details was obtained from the patient/parent/guardian/relative of the patient.

